# Regulation of CDK9 Activity by Phosphorylation and Dephosphorylation

**DOI:** 10.1155/2014/964964

**Published:** 2014-01-12

**Authors:** Sergei Nekhai, Michael Petukhov, Denitra Breuer

**Affiliations:** ^1^Center for Sickle Cell Disease, Department of Medicine, Howard University, 520 W Street, N.W. Washington, DC 20059, USA; ^2^Division of Molecular and Radiation Biophysics, Petersburg Nuclear Physics Institute, Gatchina 188350, Russia; ^3^Department of Biophysics, St. Petersburg State Polytechnical University, Polytechnicheskaya Street 29, St. Petersburg 195251, Russia

## Abstract

HIV-1 transcription is regulated by CDK9/cyclin T1, which, unlike a typical cell cycle-dependent kinase, is regulated by associating with 7SK small nuclear ribonuclear protein complex (snRNP). While the protein components of this complex are well studied, the mechanism of the complex formation is still not fully understood. The association of CDK9/cyclin T1 with 7SK snRNP is, in part, regulated by a reversible CDK9 phosphorylation. Here, we present a comprehensive review of the kinases and phosphatases involved in CDK9 phosphorylation and discuss their role in regulation of HIV-1 replication and potential for being targeted for drug development. We propose a novel pathway of HIV-1 transcription regulation via CDK9 Ser-90 phosphorylation by CDK2 and CDK9 Ser-175 dephosphorylation by protein phosphatase-1.

## 1. Introduction

Complete eradication of HIV-1 infection requires novel approaches to induce integrated HIV-1 provirus which is not affected by the existing antiretroviral drugs and which is rebound upon the termination of the antiretroviral therapy [1]. HIV-1 latency can be a result of several factors such as deficiency of HIV-1 transcriptional activator protein (Tat) or cellular transcriptional activators, transcriptional interference with cellular promoters, unfavorable integration site, epigenetics, and likely other factors [2]. Thus, better understanding the mechanisms of HIV-1 transcription activation is important for the design of novel therapeutics aimed at induction as well as inhibition of HIV-1 transcription. Here, we review the kinases and phosphatases involved in the activation of CDK9/cyclin T1 and discuss how these enzymes can be potentially used to inhibit or activate HIV-1 for the development of future therapeutic interventions for the treatment of HIV-1 infections.

## 2. Activation of HIV-1 Transcription by P-TEFb

**Figure 1:**
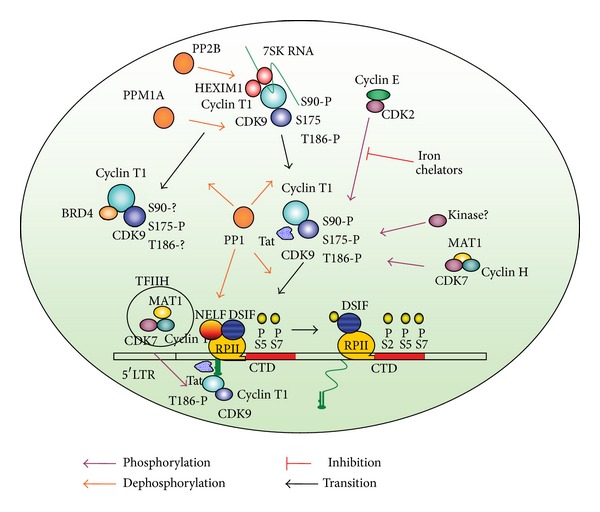
Schematic representation of the HIV-1 transcription regulation by CDK9 phosphorylation and dephosphorylation. The figure depicts a network of Tat-interacting host cell factors that affect CDK9 phosphorylation. Arrows indicate phosphorylation (violet), dephosphorylation (orange), and transition of complex P-TEF and transcription complexes (black). CDK2 phosphorylates CDK9 Ser-90. Iron chelators reduce cellular activity of CDK2/cyclin E and inhibit HIV-1 transcription (indicated by red line). CDK7 phosphorylates CDK9 Thr-186. Dephosphorylation of Thr-186 by PPM1A or PP1 facilitates dissociation of CDK9/cyclin T1 from large P-TEFb complex and recruitment of CDK9/cyclin T1 by Tat or BRD4. BRD4 preferentially binds Ser-175-phosphorylated CDK9. Dephosphorylation of Ser-175 by PP1 activates CDK9 kinase activity and activates HIV-1 transcription. Recruitment of CDK9/cyclin T1 by Tat to TAR RNA leads to phosphorylation of NELF which dissociates and also phosphorylation of DSIF and RNAPII CTD Ser-2 residues, which is facilitated by CTD Ser-7 phosphorylation. CDK7 as part of TFIIH phosphorylates CTD Ser-5 and possibly Ser-7 residues. CDK7 also phosphorylates CDK9 Thr-186 and maintains CDK9 Thr-186 phosphorylation during the elongation of transcription.

HIV-1 transcription is activated by HIV-1 Tat protein that binds to the bulge of TAR RNA, a hairpin-loop structure located at the 5′-end of all nascent HIV-1 transcripts, and recruits CDK9/cyclin T1, a component of positive transcription elongation factor b (P-TEFb) to the HIV-1 promoter (reviewed in detail in [3]; see also illustration in Figure 1). During the transcription initiation, TFIIH-associated CDK7/cyclin H phosphorylates Ser-5 within ^1^YSPTSPS^7^ heptapeptide sequence repeated 52 times in the C-terminal domain (CTD) of RNAPII [4]. The Ser-5 phosphorylated RNAPII accumulates at 20–40 nucleotides (nt) downstream of the transcription start site, partly owing to the actions of the negative-acting elongation factor complex, NELF, and the DRB-sensitivity inducing complex, DSIF [5]. Recently TFIIH-associated CDK7 was shown to phosphorylate also CTD Ser-7 residues [6], which may prime Ser-2 phosphorylation by P-TEFb [7]. Recruitment of P-TEFb to the HIV-1 promoter located within U3-R-U5 region of the 5′ LTR is facilitated by Tat which targets CDK9/cyclin T1 to TAR RNA where cyclin T1 binds to the G-rich loop of TAR RNA [8]. Recruitment of CDK9/cyclin T1 promotes transcription elongation by RNA polymerase II (RNAPII) which otherwise is paused after the synthesis of TAR RNA [9]. Release of RNAPII from the pause by P-TEFb complex is accompanied by mRNA capping and loss of NELF [10]. P-TEFb triggers elongation of RNA polymerase II (RNAPII) transcription by phosphorylating the negative elongation factor (NELF) and the DRB-sensitivity inducing complex (DSIF/Spt4/Spt5), thus promoting the release of NELF and also phosphorylating Ser-2 residues in RNAPII CTD (reviewed in [9]). Upon the dissociation of NELF, DSIF becomes a positive elongation factor [11] and increased the processivity RNAPII [12]. Although P-TEFb travels with the elongation complex, its CTD kinase activity is no longer required once the complex is released from the pause [10].

Because of the importance of P-TEFb, regulation must be maintained to insure proper function. Phosphorylation and dephosphorylation of specific sites on CDK9 must occur throughout the transcription process and must be tightly controlled. This review focuses on the regulation of CDK9 through phosphorylation modifications.

## 3. Composition of P-TEFb and Its Role in HIV-1 Transcription Activation

P-TEFb is a heterodimer consisting of cyclin-dependent kinase 9 (CDK9) and one of the C-type cyclins T1, T2a, or T2b of which cyclin T1 is the most abundant partner [10, 13]. Cyclin K which was originally thought to be a minor cyclin of CDK9 [14] is now recognized to be a cyclin partner for CDK12 and CDK13 [15, 16]. Only cyclin T1 protein efficiently forms a complex with HIV-1 Tat bound to TAR RNA [17]. This is due to the presence of a cysteine residue in cyclin T1 at position 261 rather than an asparagine found in cyclin T2a and T2b proteins. Cyclin T1 with mutated Cys-261 binds to Tat but is unable to recruit Tat to TAR RNA. Interaction of Tat with TAR RNA and cyclin T1 is dependent on zinc ions which again requires the presence of Cys-261 [18–20].

There are two functional isoforms of CDK9: a 42 kDa form predominately expressed in spleen, thymus, and testis and a 55 kDa form that is expressed in various tissues but at significantly lower levels that is the 42 Kd isoform [21, 22]. All cyclins associate with both isoforms of CDK9 and exhibit kinase activity towards CTD in RNAPII [22]. Roughly half of P-TEFb is in an inactive large complex bound by 7SK snRNA [23, 24] and several additional proteins including hexamethylene bisacetamide-inducible protein 1 (HEXIM1) [25, 26], La-related LARP7 protein [27–30], and methylphosphatase capping enzyme MePCE [31–33]. The lower molecular weight form of P-TEFb consists of CDK9 and cyclin T1 and is enzymatically active [23, 24]. The high molecular weight P-TEFb is inactive because of the HEXIM1 that introduces its inhibitory PYNT sequence into the active site of CDK9 [34].

The high molecular weight P-TEFb complex serves as a source of CDK9/cyclin T1 for the recruitment by HIV-1 Tat [35]. In stress-induced cells, the CDK9/cyclin T1 complex dissociates from the 7 SK RNA/HEXIM1 protein and then binds BRD4 and forms a transcriptionally active small complex that is recruited to various cellular promoters [36, 37]. Despite the inactivity of large P-TEFb complex *in vitro*, the large complex, and not the small complex, is important for the activation of HIV-1 transcription as HIV-1. Tat competes with HEXIM1 for binding to cyclin T1 promoting dissociation of P-TEFb from the large complex [38, 39]. HIV-1 Tat protein was also shown to recruit large P-TEFb complex to HIV-1 promoter where TAR RNA was able to displace 7 SK RNA and activate P-TEFb [40].

Tat also facilitates the formation of super elongation complex (SEC) containing active P-TEFb and additional elongation factors and transcription coactivators [41, 42]. These factors include AFF4, ENL, AF9, and elongation factor ELL2 [42]. The AFF4 protein emerges as the central scaffold that recruits other factors through direct interactions. AFF4 binds cyclin T1, ELL2, and ENL or AF9 acting as a bridge that links this complex to P-TEFb [43]. Through the bridging functions of Tat and AFF4, P-TEFb and ELL2 combine to form a bifunctional elongation complex that greatly activates HIV-1 transcription [42, 44]. Without Tat, AFF4 can mediate the ELL2-P-TEFb interaction, albeit inefficiently. Tat overcomes this limitation by bringing more ELL2 to P-TEFb and stabilizing ELL2 in a process that requires active P-TEFb [42].

## 4. CDK9 Phosphorylation

**Figure 2:**
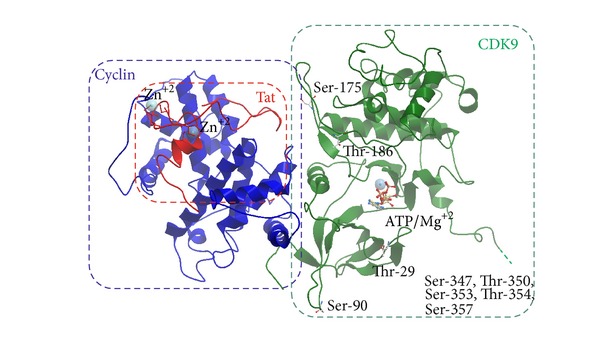
Structure of CDK9/cyclin T1/Tat complex (model based on PDB 3MIA). Backbone representation of CDK9 residues present in original PDB structure is shown in green color, cyclin T1—in violet, and Tat—in red. Position of Thr-29, Ser-175, Thr-186, and C-terminal Ser/Thr cluster is indicated. Also shown are ATP-binding site and Zn ions that facilitate Tat-cyclin T1 interaction.

Activity of P-TEFb is dependent upon phosphorylation of several serine and threonine residues and we will discuss this below and which are shown in the CDK9/cyclin T1/Tat model in Figure 2.

### 4.1. C-Terminal CDK9 Phosphorylation

A cluster of CDK9's C-terminal residues (Ser-347, Ser-353, and Ser-357; Thr-350 and Thr-354) is autophosphorylated and this phosphorylation is important for the binding of CDK9/cyclin T1 to TAR RNA [22, 45, 46]. This was evidenced by the inability of enzymatically active C-terminally truncated CDK9 to bind TAR RNA [45]. Recombinant CDK9/cyclin T1 that was autophosphorylated *in vitro* was efficiently dephoshorylated by protein phosphatase 2A (PP2A) but not the protein phosphatase-1 (PP1) [47]. Also treatment with PP2A prevented the binding of CDK9/cyclin T1 to Tat and TAR RNA *in vitro* [47]. These observations suggested that PP2A may target the C-terminus of CDK9 and potentially control the interaction of P-TEFb with TAR RNA. Autophosphorylation of CDK9 associated with the preinitiation complex *in vitro* was inhibited by TFIIH [48]. PP2A was later found to be essential for basal HIV-1 transcription *in vitro*, as PP2A depletion inhibited basal HIV-1 transcription and the add-back of PP2A to the depleted extracts restored the transcription [49]. The PP2A target was thought to be Thr-29 (see below), but this was not proven with certainty and the effect was not reproduced *in vivo*. In an early study, we observed a mild induction of basal but not Tat-induced HIV-1 transcription by PP2A-inhibitory low concentrations of okadaic acid [50]. We also observed induction of basal and not the Tat-activated HIV-1 transcription with the overexpression of LIS1 protein which we found to bind and inhibit PP2A *in vitro* [50] and interact with HIV-1 Tat protein [51]. Collectively, these studies indicate that PP2A may regulate basal HIV-1 transcription and also control the interaction of P-TEFb with TAR RNA by dephosphorylation the C-terminus of CDK9.

### 4.2. N-Terminal Thr-29 Phosphorylation

Phosphorylation of CDK9's Thr-29 was shown to be induced by HTLV-1 Tax protein [52]. CDK9/cyclin T1 is recruited to chromatinized HIV-1 LTR and other promoters by BRD4 [36, 37]. This BRD4 recruitment was linked to the phosphorylation of CDK9 Thr-29 and the requirement for PP2A dephosphorylation by John Brady's group [49]. However, Qiang Zhou's group reported that CDK9 is needed to be phosphorylated on Ser-175 to bind to BRD4 [36], which was recently confirmed by Jonathan Karn's group [53] (see detailed discussion below). CDK9's Thr-29 is homologous to Thr-15 on CDK2, in which phosphorylation inhibits CDK2 activity [54, 55]. Indeed phosphorylation of Thr-29 was shown to inhibit CDK9 activity and HIV-1 transcription [49]. However, we failed to detect CDK9 Thr-29 phosphorylation in cells treated with okadaic acid that inhibited both PP2A and PP1 using a combination of Hunter 2D thin layer electrophoresis and high resolution mass spectrometry which only showed phosphorylation of C-terminal Ser/Thr cluster and Ser-175 of which only Ser-175 phosphorylation was induced by okadaic acid [56]. Thus, CDK9 Thr-29 phosphorylation may not be controlled by PP2A and needs to be further validated to clarify its role during HIV-1 transcription.

### 4.3. CDK9's T-Loop Thr-186 Phosphorylation

CDK9 T loop contains several phosphorylation sites, including Ser-175 and Thr-186 (Figure 2). Phosphorylation of the conserved Thr-186 is necessary for the enzymatic activity of CDK9 [57, 58]. Also the association of CDK9/cyclin T1 with 7SK RNA snRNP requires CDK9's Thr-186 phosphorylation [22, 57, 58]. In CDKs, phosphorylation of the T-loop triggers major conformational changes that open ATP binding pocket and a substrate binding site making CDKs fully active as a kinase [59].

Recently, chemical-genetic analysis using selective chemical inhibition of CDK7 showed that it is solely responsible for CDK9 Thr-186 phosphorylation and for P-TEFb-dependent CTD Ser-2 phosphorylation and histone H2B ubiquitylation *in vivo* [60]. Thus, CDK7/cyclin H which was previously identified as CDK-activating kinase (CAK) for the CDKs involved in the cell cycle such as CDK1, 2, and 4 also functions as CAK for CDKs involved in the regulation of transcription that include CDK8, 9, 12, and 13 (reviewed in [61]). Global analysis of kinases that may phosphorylate CDK9 T-loop using siRNA identified Ca(2+)/calmodulin-dependent kinase 1D (CaMK1D) knock down by siRNA decreased Thr-186 phosphorylation [62]. Accordingly, small molecule inhibition of Ca(2+) signaling pathway decreased Thr-186 phosphorylation and inhibited Tat-induced HIV-1 transcription but not Tax-mediated HTLV-1 transcription [62]. Because Tax-mediated transcription is driven by P-TEFb, it remains to be further investigated why only HIV-1 transcription was affected.

CDK9 Thr-186 phosphorylation can also be controlled by cellular phosphatases. Our studies for the past decade focused on PP1 that we initially observed to stimulate Tat-dependent HIV-1 transcription *in vitro* [63]. When we overexpressed one of the major nuclear regulatory subunits of PP1, NIPP1 (nuclear inhibitor of PP1), we observed PP1-specific inhibition of HIV-1 transcription and viral replication [64]. We noticed that Tat contains a sequence that is similar to a conserved PP1-binding RVxF motif and showed that this motif mediates Tat binding to PP1 *in vitro* and *in vivo* and that mutation of residues in the PP1 binding motif (V36A and F38A) prevented Tat from inducing HIV-1 transcription [65]. CDK9 that was phosphorylated in cultured cells spiked with (^32^P)orthophosphate and treated with okadaic acid was efficiently dephophosphorylated *in vitro* by PP1 but not PP2A [47] in contrast to the *in vitro* autophosphorylated CDK9 that was dephosphorylated by PP2A and not by PP1 [47]. These results suggested that PP1 may control CDK9 phosphorylation during HIV-1 transcription. Indeed, the alpha catalytic subunit of PP1, PP1*α*, was shown to act cooperatively and sequentially with (Ca^2+^)-calmodulin-protein phosphatase 2B (PP2B) in UV irradiated or hexamethylene bisacetamide (HMBA)-treated cells in which P-TEFb is released from 7SK snRNP complex [66]. While PP2B induces a conformational change in 7SK snRNP, PP1*α* dephosphorylates CDK9 Thr-186 and facilitates the releases of P-TEFb from 7SK snRNP [66]. CDK9 remained dephosphorylated while associated with BRD4 and being recruited to the preinitiation complex, where it can be reactivated by TFIIH-associated CDK7. In accordance with this study, we observed increased CDK9 Thr-186 phosphorylation in the cells that stably expressed a PP1-inhibitory peptide, the central domain of NIPP1, and also observed increased association of CDK9/cyclin T1 with 7SK RNA [67]. Stable expression of cdNIPP1 disrupted the interaction between Tat and PP1 and inhibited HIV-1 transcription [67], suggesting that a balance needs to be maintained between phosphorylation and dephosphorylation of CDK9 Thr-186 and that shifting this balance toward the phosphorylation is inhibitory for HIV-1 transcription. Expression of cdNIPP1 in a physiologically relevant manner as part of the HIV-1 genome in place of *nef* potently inhibited HIV-1 replication [67], further suggesting that PP1-targeted inhibitors can be of use as potential anti-HIV-1 therapeutics. While PP1 is a candidate for Thr-186 dephosphorylation, we also found that it dephosphorylates CDK9 Ser-175 (see below). An additional CDK9 Thr-186 phosphatase, magnesium-dependent protein phosphatase 1A (Formerly 2C) (PPM1A) was identified using a phosphatase expression library and Thr-186-phosphospecific antibodies [68]. PPM1A overexpression decreased Thr-186 phosphorylation and siRNA-mediated depletion increased it, and P-TEFb and PPM1A also coprecipitated together suggesting that CDK9 can be a physiological substrate for PPM1A [68]. A more recent study showed that CDK9's Thr-186 phosphorylation is decreased in resting CD4(+) T cells which are non permissive for HIV-1 replication and that this decrease correlated with the abundance of PPM1A and limited recruitment of CDK9 to the large P-TEFb complex [69]. This finding further supports the necessity of the large complex for HIV-1 transcription and the critical role of PPM1A in the HIV-1 suppression in resting T cells. Because PPM1A expression was not changed upon T cells activation [69], yet unknown regulatory factors may be involved in the regulation of the PPM1A activity.

### 4.4. CDK9's T-Loop Ser-175 Phosphorylation

Once CDK9/cyclin T1 dissociates from the large P-TEFb complex, CDK9 may become phosphorylated on Ser-175. While Thr-186 dephosphorylation totally inhibited CDK9/cyclin T1 kinase activity, no difference was found in the kinase activities of CDK9 S175A and CDK9 S175D mutants by David Price and colleagues [58]. In contrast, Qiang Zhou and his group showed that CDK9 S175A mutant was inactive, while CDK9 S175D mutant was active as kinase [36, 57]. They also showed that Ser-175 phosphorylation promotes binding of CDK9/cyclin T1 to Brd4 [36]. It was suggested that phosphorylation of Ser-175 may cause a conformational change in CDK9, allowing BRD4 to bind to cyclin T1 [36]. In our recent study, we found that dynamic inhibition of PP1 led to exclusive phosphorylation of CDK9 Ser-175 as determined by a combination of Hunter 2D peptide mapping and LC-MS analysis *in vivo* [56]. Inhibition of PP1 led to the inhibition of CDK9 activity and reduction of RNAPII phosphorylation *in vitro *and *in vivo* [56]. We found that CDK9 S175A mutant was enzymatically active and induced HIV-1 transcription [56]. Interestingly, while CDK9 S175D mutant was less active as kinase, it more readily formed small P-TEFb complex especially when PP1 was inhibited. Thus we concluded that PP1 activates HIV-1 transcription by dephosphorylating CDK9 Ser-175 residue. We also noticed that Ser-175 phosphorylation activity was much more abundant than Thr-186 activity in cell extracts suggesting that CDK9 activity may be naturally suppressed through the overphosphorylation of Ser-175. A recent study by Jonathan Karn's group showed that activation of T cells through T-cell receptor (TCR) or phorbol ester (PMA) signaling strongly induced phosphorylation of the CDK9 Ser-175 [53]. Based on the molecular modeling they proposed that phosphorylated Ser-175 forms a hydrogen bond with Tat Lys-14 strengthening the binding of Tat to CDK9/cyclin T1 and promoting HIV-1 transcription activation [53]. Also in accordance with the early observations, CDK9 S175A mutation was found to abolish the binding to BRD4 [53]. Also, in accord with our observations, CDK9 S175A mutant was found to greatly induce Tat-dependent latent HIV-1 reactivation, which Jonathan Karn and his coworkers attributed to the inability of this mutant to bind BRD4 while being able to bind to Tat [53]. They also found that CDK9 phosphorylated at Ser-175 is excluded from the 7SK RNP complex [53], which parallels our earlier observation that CDK9 S175D phosphomimetic mutant was found in small P-TEFb complex [56]. Thus, this latest study points to Ser-175 as an important target for HIV-1 reactivation and potential development of small molecule therapeutics.

We recently developed PP1-targeted small molecules that were modeled to fit the RVxF-accommodating cavity on PP1 [70]. We screened virtually about 300,000 compounds and then physically screened about 1000 compounds and identified one hit compound, 1H4, which inhibited HIV-1 transcription and replication at noncytotoxic concentrations [70]. 1H4 prevented PP1-mediated dephosphorylation of a substrate peptide containing an RVxF sequence *in vitro*, disrupted the association of PP1 with Tat in cultured cells, and prevented the translocation of PP1 to the nucleus [70]. We are currently in the process of refining the hit compound and also developing PP1-targeted compounds for the activation of latent provirus.

### 4.5. CDK9's Ser-90 Phosphorylation

We and others have shown earlier that HIV-1 transcription is activated by Tat in the G1 phase, but not in the G2 phase [71, 72]. Thus we hypothesized that Tat might engage a cell cycle regulatory kinase, which we showed to be CDK2/cyclin E [73, 74]. HIV-1 is inhibited in the CDK2 knockdown cells [75] and also in macrophages differentiation from induced pluripotent stem cells with CDK2 knockdown [76], further suggesting that CDK2 is important for HIV-1 transcription and replication. The functional link between CDK2 and CDK9 was found when we analyzed inhibition of HIV-1 by iron chelators. HIV-1 transcription was inhibited in T cells treated with iron chelators *311* and ICL670 which also inhibited the cellular activity of CDK2 and CDK9 [77]. More potent di-2-pyridylketone thiosemicarbazone-based tridentate iron chelators inhibited HIV-1 transcription and virus replication at much lower concentrations than 311 or ILC670 and also inhibited CDK2 and CDK9 activity [78]. While the mechanism of CDK2 inhibition is not yet clarified, it is likely to include induction of p21 through the upregulation of hypoxia-induced factor 1*α* (HIF-1*α*) as iron depletion removes iron from prolyl hydroxylase and increases HIF-1*α* and HIF-2*α* protein levels mimicking the effect of hypoxia [79]. We analyzed CDK9 phosphorylation by CDK2 *in vitro* and identified a motif (^90^SPYNR^94^) that represented a consensus CDK2 phosphorylation site and which was efficiently phosphorylated [80]. Phosphorylation of CDK9 on Ser-90 was detected with phosphospecific antibodies and it was reduced after the knockdown of CDK2. CDK9 S90A mutant association with the large P-TEFb complex was reduced and its overexpression inhibited HIV-1 transcription. In contrast, CDK9 S90D mutant showed unchanged association with large and small P-TEFb complexes and induced Tat-dependent HIV-1 transcription. However, the phosphomimetic S90 showed an overall decrease in CDK9 expression [80] suggesting that a phosphorylation/dephosphorylation balance must be maintained to form the large and small P-TEFb complexes. Molecular modeling showed that Ser-90 of CDK9 was located on a flexible loop exposed to solvent, suggesting that it might be undergoing phosphorylation (also seen on Figure 2). Thus our recent studies identified a novel regulatory phosphorylation site on CDK9 that may be targeted for activation or inhibition of HIV-1 transcription.

## 5. Conclusion

Phosphorylation and dephosphorylation of specific sites on CDK9 occurs throughout the transcription process and must be tightly controlled. Modifications at certain threonine/serine residues determine whether CDK9 associates with large P-TEFb complex to become available for recruitment and whether the dissociation of the large complex will efficiently occur. Phosphorylation of Thr-186 in the T-loop of CDK9 and Ser-90 which lies outside of the T-loop determines whether CDK9 remains sequestered in the inactive large complex and also whether the kinase is enzymatically active once it is dissociated from 7SK snRNP. Dephosphorylation at either of these sites leads to destabilization of the large complex and release of CDK9/Cyclin T1, thereby restricting Tat recruitment to HIV-1 LTR. Modifications in the CDK9's C-terminus allow for cyclin T1 : TAR binding and dephosphorylation at these sites prevents binding toTAR RNA. In contrast, phosphorylation at Thr-29 or Ser-175 residues inhibits CDK9. Thus the emerging picture of CDK9 regulation through phosphorylation seems to be complex and in part parallels what is known for other CDKs. The recently identified CDK9-targeted kinases, CDK7, CDK2, and phosphatases, PP1, and PPM1A will likely emerge as novel targets for anti-HIV-1 and cancer therapeutics.
